# Oral Dermoid Cyst in the Mouth Floor: A Case Report and 5-Year Review of the Literature

**DOI:** 10.1155/crid/8851087

**Published:** 2025-08-30

**Authors:** Marco Rossi, Claudia Manera, Francesco Saverio Ludovichetti, Christian Bacci

**Affiliations:** ^1^Department of Maxillofacial Surgery, ULSS2, Vittorio Veneto Hospital, Vittorio Veneto, Treviso, Italy; ^2^Department of Neurosciences–Dentistry Section, Padova University, Padova, Italy

**Keywords:** dermoid cyst, head tumors, oral cavity, radiological assessment

## Abstract

**Aim:** The aim of the study is to present a case of a dermoid cyst in the oral floor of an 80-year-old female patient and discuss its clinical presentation, diagnostic approach, and treatment.

**Methods:** A retrospective case study was conducted at the Department of Maxillofacial Surgery, Aulss 2 Marca Trevigiana. The patient, with a medical history of hypertension, presented with a swelling in the oral floor. A clinical examination was followed by a facial CT scan with and without contrast medium to evaluate the cystic lesion. The patient underwent surgical excision under general anesthesia, with subsequent histopathological analysis.

**Results:** The CT scan revealed a round, homogeneous, hyperdense formation with no enhancement after contrast administration. Surgical excision was performed using an intraoral approach, and histopathology confirmed the diagnosis of a dermoid cyst with an epithelial lining of predominantly squamous type. The patient experienced no complications, and follow-up at 9 days and 2 months showed complete recovery.

**Conclusion:** A dermoid cyst of the oral cavity, though rare in adults, should be considered in the differential diagnosis of oral floor swellings. Early diagnosis through clinical examination and imaging, followed by surgical excision, provides an effective and curative treatment.

## 1. Introduction

The term “dermoid cyst” (DC) is often used generically to describe three distinct histological entities: the oral epidermoid cyst (EC), the DC proper, and the teratoid cyst (TC). Since all three of these cysts are characterized by an embryological developmental defect, they are collectively referred to as dysontogenic cysts. The oral DC is a rare developmental cystic malformation, primarily found in children and young adults, and is congenital in 15% of cases. It typically develops in the oral floor, in a median position, and rarely in a lateral or other locations [1, 2].

Clinically, it presents as a nontender swelling of the oral floor, with normal color, increased consistency, and slow growth. The size can vary from a few millimeters to several centimeters, potentially impairing phonation, respiration, and feeding capacity for the patient. Like all cystic lesions, it may become infected, leading to an abscess that tends to fistulize either intraorally or extraorally [3].

The differential diagnosis includes various pathological entities that can cause swelling of the oral floor, such as the lymphoepithelial cyst, salivary gland pathologies due to obstruction (sialolithiasis or ranula), and benign or malignant neoplastic conditions such as squamous cell carcinoma of the oral cavity [4].

Instrumental examinations useful for differential diagnosis include computed tomography (CT) of the facial mass with and without contrast medium (CM), magnetic resonance imaging (MRI) of the head and neck with and without CM, and ultrasonography of the salivary glands [5–7].

The diagnosis of DC is clinical, instrumental, and histopathological. The treatment is surgical and curative.

Here, we present a clinical case of an oral floor DC in an 80-year-old female patient, who was referred to our attention at the UOSD of Maxillofacial Surgery, Aulss 2 Marca Trevigiana, Veneto, Italy. The patient gave her consent for publication.

## 2. Case Presentation

An 80-year-old female patient presented to our attention at the Department of Maxillofacial Surgery, Aulss 2 Marca Trevigiana, Veneto, Italy. The patient reported swelling in the oral floor region with a sensation of “fullness” in both the oral floor and the tongue.

Her medical history revealed hypertension, for which she was undergoing pharmacological treatment with rosuvastatin + ezetimibe, perindopril, and indapamide. Other medications included a vitamin D supplement and omeprazole. She denied any known allergies. The patient was a user of upper and lower removable complete dentures.

**Figure 1:**
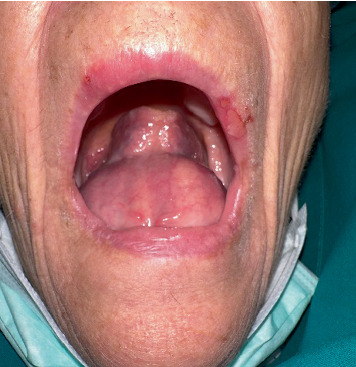
Large and prominent submucosal swelling in the floor of the mouth.

Upon intraoral examination, a prominent submucosal swelling of the anterior oral floor was noted, with a round appearance, soft consistency, and normal color. This swelling occupied the entire anterior oral floor and displaced the mobile tongue posteriorly and superiorly, as shown in Figure 1.

**Figure 2:**
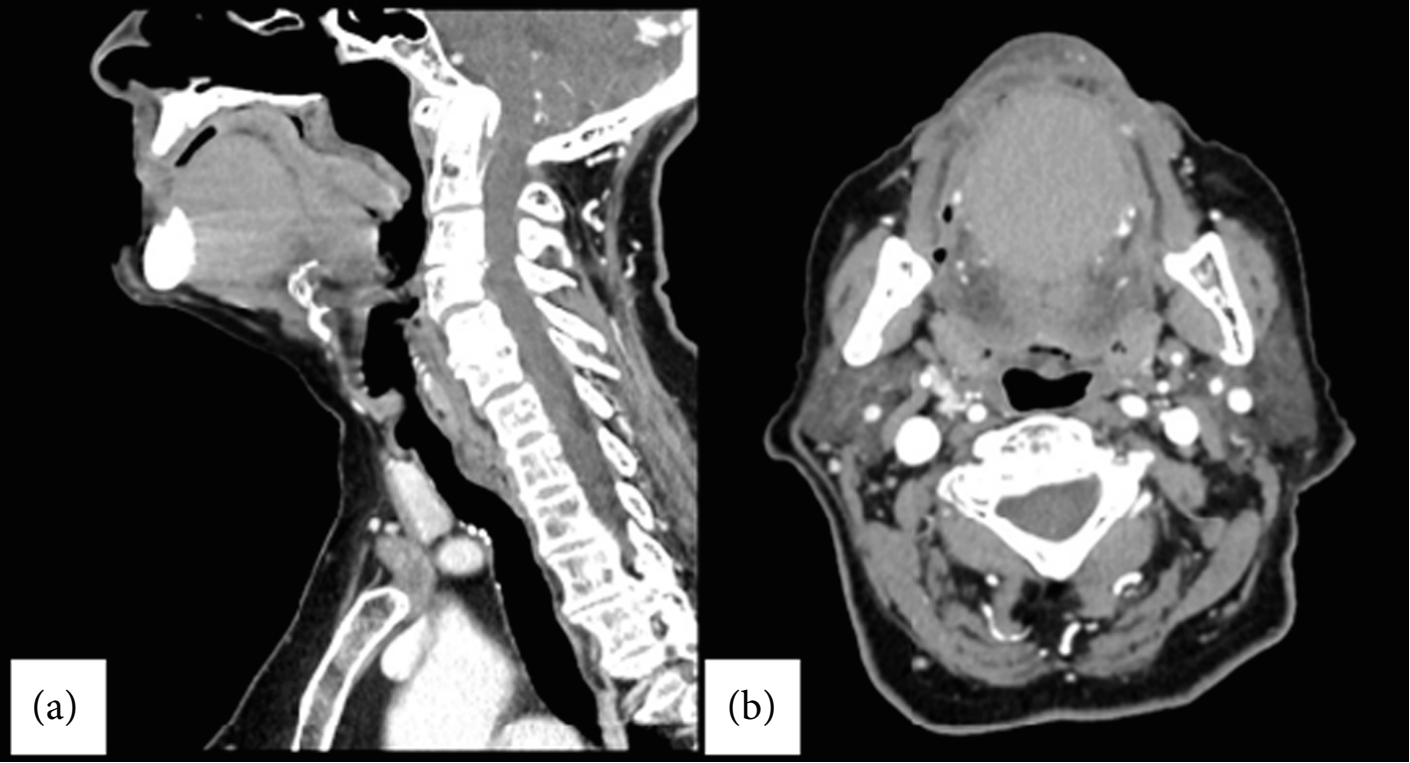
CT scan in (a) sagittal and (b) axial sections that highlight a rounded neoformation located at the level of the oral floor, not absorbing the contrast medium.

The patient subsequently underwent further diagnostic radiological assessment with a facial CT scan with and without CM. This examination identified a large, roughly round formation measuring approximately 4.5 × 4 cm in the axial plane and extending approximately 4 cm craniocaudally, with a small extension between the geniohyoid muscles. The formation exhibited homogeneous hyperdense content without enhancement after intravenous administration of CM, as seen in Figure 2.

The clinical and radiological suspicion suggested a cystic formation with a high protein content, potentially a ranula of the oral floor.

In agreement with the patient and after obtaining informed consent, a procedure for excision of the swelling followed by histopathological investigation was scheduled. The surgery was performed under general anesthesia in an in-patient setting. The procedure was completed without complications.

Electrocautery was used, and blunt dissection was carried out in layers to isolate the lesion's capsule. The lesion was completely excised, and a dense, yellowish liquid content was observed. Hemostasis was achieved with the application of absorbable sutures and by using oxidized regenerated cellulose.

**Figure 3:**
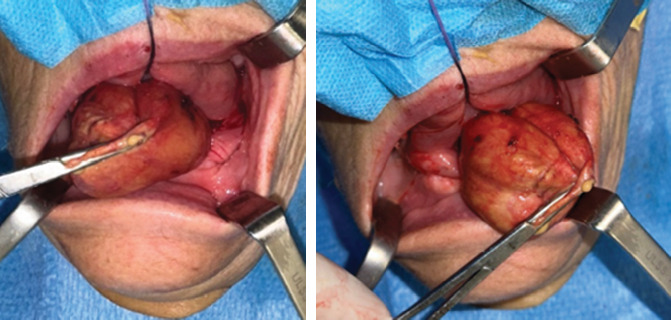
Enucleation procedure of the cystic lesion from the floor of the oral cavity.

**Figure 4:**
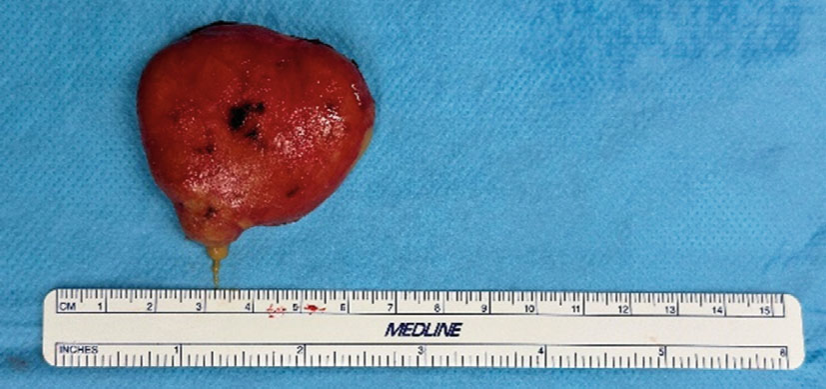
Totally enucleated lesion.

Figures 3 and 4 show the surgical procedure of enucleation.

The histopathological findings were consistent with a DC of the oral cavity. Sections of the cyst showed an epithelial lining, predominantly squamous in type, without significant nuclear–cytoplasmic atypia, and mild fibrosis of the wall.

**Figure 5:**
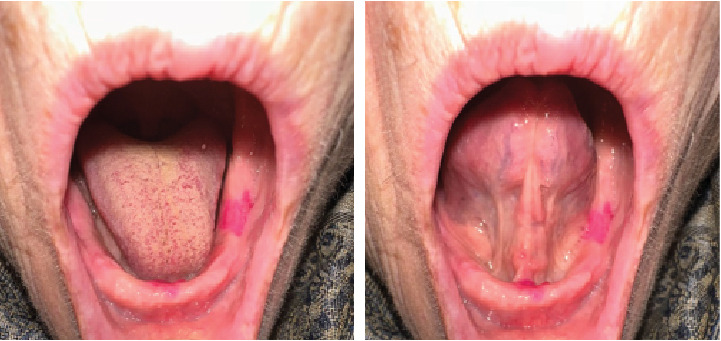
Complete restoration of the anatomy of the oral floor, at the 2-month follow-up.

The patient was reevaluated 9 days postoperation for suture removal and at 2 months. During the 2-month follow-up, a complete restoration of the normal oral floor anatomy was observed, as shown in Figure 5.

## 3. Discussion

The term “DC” refers to three distinct histological entities resulting from an embryological developmental defect of unknown pathogenesis: the oral EC, the DC proper, and the TC. These cysts are collectively described as dysontogenic cysts, and their incidence ranges from 1.6% to 7% in the head and neck region [1, 2].

The EC is lined by epidermis-like epithelium and does not contain any dermal appendages in the cyst wall. The DC differs from the EC by the presence of associated structures such as sebaceous elements, sweat glands, and hair follicles within the cyst wall. The TC is a cystic form of teratoma containing ectodermal, mesodermal, and endodermal derivatives within the cyst wall; it is also referred to as a complex cyst [1, 4]. Stratified squamous epithelium is the predominant epithelial lining of these cystic lesions. Additionally, Pacini bodies may be unusual findings in DCs [8]. In our case, histological analysis revealed an epithelial lining predominantly of squamous type, with no significant nuclear–cytoplasmic atypia and mild fibrosis of the wall. The findings were therefore suggestive of a DC of the oral cavity.

The DC is a rare benign tumor of the oral cavity, representing less than 0.01% of oral cavity lesions and 0.29% of head and neck tumors in children [9, 10]. However, it is predominantly found in the pediatric population, with rare cases in adults [11–13]. The pathology and pathogenesis of DCs are described in greater detail by Rapidis et al. [14]. DCs can develop anywhere in the body, but approximately 6.5% occur in the oral floor, causing, in some cases, dysphonia, dysphagia, and dyspnea [15].

A literature review was conducted on PubMed using the keywords “dermoid cyst” and “oral cavity,” and 19 case reports of DCs were selected based on the available abstracts from the period 2020–2025. Papers without available abstracts were excluded.

The case series found is listed in Table 1.

In a retrospective cross-sectional study conducted by Cunha et al., it was reported that the DC predominantly localizes in the oral floor (*n* = 14, 45.2%), with a higher prevalence in females (*n* = 17, 53.1%), with a mean age of 34.6 ± 21.6 years [27]. In a retrospective review conducted by Oluleke et al. of 14 patients with sublingual DCs, managed over an 8-year period from January 2010 to December 2017, 8 males (57.1%) and 6 females (42.9%) were reported. The male-to-female ratio was 1.3:1, with an age range from Day 1 to 25 years [28]

DCs rarely develop at the ventral tongue. Most are located in the median position (52% of cases sublingual and 26% submental), 16% occupy one to three spaces (submental, sublingual, and submandibular), and only 6% are localized exclusively in the submandibular space [4]. A rare case of a DC located in the palate was reported by Khalifeh et al. [26], as well as a case of DC localization in the upper lip and uvula [13, 21]. In some cases, the DC may coexist with heterotopic oral gastrointestinal cyst, a rare entity occurring in infants and children, with a predilection for males [29–31]. In our case, the DC identified occupied the oral floor of an 80-year-old female patient.

DCs must be differentiated from various pathological entities that can cause swelling in the head and neck region, such as embryological anomalies (thyroglossal duct cyst, branchial cleft cyst, cystic hygroma, lymphoepithelial cysts), infections (acute bacterial infection/cellulitis of the oral floor, sialadenitis of the sublingual or submandibular gland, and viral lymphadenitis), salivary gland pathologies (mucoceles and ranula), and both benign and malignant neoplastic pathologies of different entities [4]. For this reason, the clinical diagnosis must be supported by instrumental diagnostics to properly plan any potential surgical excision. Like many benign tumors of the oral cavity, DCs may only be identified on cross-sectional images such as CT and MRI. Therefore, CT and MRI play an important role in diagnosing these unusual lesions [5, 6]. Specifically, in our case, the patient underwent a facial CT scan with and without CM. The examination revealed a large, grossly rounded formation with a small extension between the geniohyoid muscles. This formation was homogeneously hyperdense and showed no enhancement after the administration of contrast material. Ultrasound imaging, using head and neck ultrasound, can also be helpful in the instrumental diagnosis [7].

The treatment of choice for head and neck DCs and ECs remains surgical excision. Oral cavity/floor of mouth cysts can be excised using either the intraoral or extraoral approach. The former is preferred for small sublingual cysts superior to the mylohyoid muscle, while the latter can be used for larger cysts and lesions inferior to the muscle [19]. In our case, an intraoral approach was chosen, and the lesion was completely enucleated using electrosurgery. The recurrence rate postexcision is rare due to the presence of a fibrous capsule, which facilitates cyst enucleation. Anyway, recurrence of a dysontogenic cyst may be secondary to a tract not identified at the time of surgery [32]. As in our case, no recurrences were observed during follow-up at 9 days and 2 months postsurgery. The literature does not specify a minimum follow-up period to identify a recurrence. Some authors suggest 1 year [33], others up to 5 years [34]. In our case, the patient is still in follow-up, and no recurrence has occurred at this time.

## 4. Conclusion

In conclusion, DCs of the oral cavity, although rare, present as benign developmental lesions that can lead to significant clinical challenges due to their location and potential complications such as dysphagia, dysphonia, or dyspnea. Their management requires a combination of clinical suspicion, instrumental diagnostics, and histopathological confirmation. The case presented in this study, involving an 80-year-old female with a DC of the oral floor, highlights the importance of early recognition and surgical intervention. Surgical excision remains the treatment of choice, with a low recurrence rate due to the cyst's fibrous capsule, as demonstrated by the patient's successful outcome following complete cyst enucleation. Given the rarity of DCs in adults, particularly in the oral cavity, clinicians must consider them in the differential diagnosis of oral floor swellings and use appropriate imaging techniques, such as CT and MRI, to guide treatment decisions.

## Figures and Tables

**Table 1:** Nineteen case reports of DCs selected based on the available abstracts in PubMed, from the period 2020–2025.

**Study**	**Year**	**Patient age**	**Sex**	**Swelling location**
Liu et al. [16]	2020	Infant	Not specified	Floor of the mouth
Matsuzaki et al. [13]	2020	50 years	M	Floor of the mouth
Robinson et al. [17]	2021	1 month	Not specified	Ventral tongue
Patel et al. [12]	2022	30 years	M	Floor of the mouth
Dwivedi et al. [18]	2022	Teenager	Not specified	Floor of the mouth
Sauer et al. [7]	2023	10 years	F	Submental
Hamada et al. [19]	2023	2 years	F	Floor of the mouth
Alhusain et al. [20]	2023	26 years	F	Left parotid mass
Harmon et al. [21]	2023	14 months	F	Uvular
Barzegar et al. [22]	2023	17 years	F	Floor of the mouth
Gleichmann et al. [5]	2023	6 years	F	Submental and sublingual
Jain et al. [9]	2024	16 years	F	Floor and left submandibular region
Szala et al. [10]	2024	13 years	M	Sublingual
Alanazi et al. [23]	2024	13 years	M	Floor of the mouth
Jangra et al. [11]	2024	32 years	F	Sublingual
Bargiel et al. [24]	2024	17 years	F	Sublingual, submental, and lingual
Safia et al. [25]	2024	12 years	M	Sublingual
Naik et al. [3]	2024	6 months	F	Floor of the mouth
Khalifeh et al. [26]	2025	2 years	F	Hard palate

## Data Availability

Data sharing not applicable to this article as no datasets were generated or analysed during the current study.
